# Abnormal Topological Organization of White Matter Structural Networks in Normal Tension Glaucoma Revealed via Diffusion Tensor Tractography

**DOI:** 10.3390/brainsci13111597

**Published:** 2023-11-17

**Authors:** Yin Wang, Linying Guo, Rong Wang, Yuzhe Wang, Fei Duan, Yang Zhan, Jingfeng Cheng, Xinghuai Sun, Zuohua Tang

**Affiliations:** 1 Department of Radiology, Eye & ENT Hospital of Fudan University, Fudan University, Shanghai 200031, Chinaduanfei0221@163.com (F.D.);; 2 Department of Radiology, Huashan Hospital of Fudan University, Fudan University, Shanghai 200040, China; 3 Department of Radiology, Zhongshan Hospital of Fudan University, Fudan University, Shanghai 200032, China; wang.yuzhe@zs-hospital.sh.cn (Y.W.);; 4 Department of Ophthalmology & Visual Science, Eye & ENT Hospital of Fudan University, Fudan University, Shanghai 200031, China; xhsun@shmu.edu.cn

**Keywords:** diffusion tensor imaging, normal tension glaucoma, structural networks, small-world properties

## Abstract

Background: Normal tension glaucoma (NTG) is considered a neurodegenerative disease with glaucomatous damage extending to diffuse brain areas. Therefore, this study aims to explore the abnormalities in the NTG structural network to help in the early diagnosis and course evaluation of NTG. Methods: The structural networks of 46 NTG patients and 19 age- and sex-matched healthy controls were constructed using diffusion tensor imaging, followed by graph theory analysis and correlation analysis of small-world properties with glaucoma clinical indicators. In addition, the network-based statistical analysis (NBS) method was used to compare structural network connectivity differences between NTG patients and healthy controls. Results: Structural brain networks in both NTG and NC groups exhibited small-world properties. However, the small-world index in the severe NTG group was reduced and correlated with a mean deviation of the visual field (MDVF) and retinal nerve fiber layer (RNFL) thickness. When compared to healthy controls, degree centrality and nodal efficiency in visual brain areas were significantly decreased, and betweenness centrality and nodal local efficiency in both visual and nonvisual brain areas were also significantly altered in NTG patients (all *p* < 0.05, FDR corrected). Furthermore, NTG patients exhibited increased structural connectivity in the occipitotemporal area, with the left fusiform gyrus (FFG.L) as the hub (*p* < 0.05). Conclusions: NTG exhibited altered global properties and local properties of visual and cognitive-emotional brain areas, with enhanced structural connections within the occipitotemporal area. Moreover, the disrupted small-world properties of white matter might be imaging biomarkers for assessing NTG progression.

## 1. Introduction 

Normal tension glaucoma (NTG) is a specific subtype of glaucoma characterized by visual field defects in the absence of elevated intraocular pressure (IOP) [1]. NTG has been likened to the “silent thief” of vision because NTG patients’ vision is eroded in the early stages without obvious clinical signs [2]. Early detection and intervention before irreversible damage occurs is critical to the prognosis of NTG. In recent years, NTG has been recognized as a neurodegenerative disease with glaucomatous damage extending from retinal ganglion cells to diffuse brain areas [3,4]. Thus, further exploration of structural brain abnormalities is expected to help in the early diagnosis and course assessment of NTG.

Diffusion tensor imaging (DTI) provides a non-invasive and efficient tool for detecting microstructural abnormalities in the white matter of the whole brain in vivo [5]. Schmidt et al. revealed axonal degeneration of the optic tract and radiation in NTG through selective tractography [6]. Furthermore, Wang et al. used atlas-based diffusion tensor analysis (ABA) to detect the whole brain and found white matter injury in visual and vision-related brain regions in NTG patients [7]. These approaches focus on glaucomatous damage to specific brain regions, while the integration of multiple brain regions supported by white matter connections is not fully understood. Graph theory is a mathematical method for analyzing complex brain networks. By identifying anatomically localized subnetworks associated with neural alterations in brain disorders, graph theory enables the study of the brain as a complex integrated network known as the human connectome rather than evaluating individual visual areas and visual bundles in isolation [8]. Previous studies have revealed the abnormal topological organization of glaucoma using graph theory [9,10,11], but the pattern of the white matter structural networks in NTG patients has not been reported.

The purpose of this study is to evaluate the integration and separation of global white matter structural networks and alterations in nodal topological properties in NTG patients using graph theory. To further identify changes in the structural connectome in NTG patients, we also applied network-based statistical analysis (NBS), a non-parametric statistical method validated in connectome analysis. Compared to edge-wise comparison, NBS analysis has a greater power to identify subnetwork differences between groups while allowing control of family error rates during connection group analysis [12]. By exploring the abnormal changes in the structural network of NTG patients, this study had the potential to yield novel insights into the neurophysiological underpinnings of the disease, thus facilitating the evaluation and monitoring of its progression.

## 2. Materials and Methods

### 2.1. Participants

A total of 65 NTG patients and 21 age- and sex-matched healthy controls were recruited from July 2019 to March 2021. Thirteen patients were excluded due to incomplete clinical information. After the MRI scan, 6 patients and 2 volunteers were excluded due to the white matter lesions diagnosed by two experienced neuroradiologists. The final study included 46 NTG patients and 19 age- and gender-matched healthy controls. All patients and healthy controls were examined by a professional ophthalmologist with 10 years of experience in glaucoma. Subjects underwent a comprehensive ophthalmic examination including retinal IOP, slit lamp microscopy, standard automated perimetry, and RNFL thickness. During the MDVF examination of the NTG patient, at least two reliable standard automated perimetry (SAPs) visual fields were recorded using the Humphrey Field Analyzer (Carl Zeiss Meditec, Dublin, CA, USA) running the 30-2 program SITA. In the measurements, the subject faces a flashing luminous sphere, responds to the sensation of a flash, and determines the sensitivity at each location in the visual field by varying the intensity of subsequent flashes. Measurements were made independently for each eye. RNFL thickness was measured using the Optic Disc Cube 200 × 200 scanning protocol of the Cirrus OCT (Carl Zeiss Meditec, Dublin, CA, USA). For OCT image acquisition, scanning laser images were focused after subjects were seated and properly positioned. The image was captured in a few seconds, during which time the patient was monitored to keep steady fixation. NTG patients were then further divided into three subgroups (mild, moderate, and severe) based on MDVF. Mild NTG (mi-NTG) is characterized by an MDVF of 0.01 to 6.00 dB. Moderate NTG (mo-NTG) is characterized by an MDVF of 6.01 to 12.00 dB. Severe NTG (se-NTG) is defined as MDVF greater than 12 dB. The exclusion criteria are as follows. (1) age < 18 years; (2) secondary glaucoma; (3) any disease that may affect the visual field; (4) clearly diagnosed central nervous system disease or psychiatric disease; and (5) any contraindication to MRI examination.

### 2.2. Acquisition Protocol

Magnetic resonance imaging was acquired using 32-channel head coils in a 3T scanner (Verio; Siemens, Erlangen, Germany). Diffusion-weighted images were acquired with a single-shot echo-planar imaging (EPI) sequence with the following parameters: repetition time = 7425 ms, echo time = 84 ms, FOV = 220 × 220 mm, matrix = 110 × 110, slice number = 50, and voxel size = 2.5 × 2.5 × 2.5 mm^3^. Diffusion-weighted images were obtained with 30 non-collinear directions (b = 1000 s/mm^2^) along with a volume without diffusion-weighted (b = 0 s/mm^2^). The high-resolution structure images were obtained with T1-weighted three-dimensional magnetization prepared rapid acquisition gradient echo (3D-MPRAGE) sequence with the following parameters: repetition time = 2530 ms, echo time = 2.34 ms, inversion time = 1100 ms, FOV = 256 mm × 256 mm, slice thickness = 1.0 mm, slices number = 192, flip angle = 9°, matrix size = 256 × 256.

### 2.3. Data Processing

The DTI images were processed fully automatically on a Linux workstation using the software pipeline for analyzing brain diffusion images (PANDA) [13]. Firstly, DTI and T1-weighted images were preprocessed with the following steps: brain extraction, eddy current distortion correction, and head motion correction. Then, T1-weighted images were segmented into gray matter, white matter, and cerebrospinal fluid using maximum posterior probability and partial volume estimation. Secondly, the diffusion tensor matrix of all participants was calculated, and the FA maps and T1 images were registered linearly using the Affine transform. Thirdly, the transformed images were normalized to ICBM-152 template via nonlinear transformation. The nearest neighbor interpolation method was used to reverse register the standard space image to the individual space of each subject. According to the method of probabilistic fiber tracking, different fiber trend maps were obtained via repeated sampling at each seed point, usually selected 5000 times. Fiber bundles and fractional anisotropy (FA) were calculated with a threshold of 0.2 and an angular threshold of 45° [14]. The nodes of the white matter structure network were divided into 90 brain regions using the AAL template. The cerebellum was excluded since it often scans incomplete and exhibits artifacts [15]. The edge of the weighted WM network was constructed based on fiber number (FN) and FA. To mitigate the impact of false connections, a threshold value for the fiber bundles was selected. A binary fiber number matrix was obtained by assigning a value of one to fiber bundles greater than or equal to 3, and zero otherwise [16]. This matrix was then multiplied by the FA values, which served as a measure of fiber integrity. The product of these matrices was used to weight the edge.

### 2.4. Network Construction

The white matter structural networks were constructed using GRETNA toolbox [17]. Small-world network parameters were used to depict global topological properties of the white matter structural networks including clustering coefficients (Cp), shortest path length (Lp), normalized Cp (γ, Gamma), normalized Lp (λ, Lambda), and small-world index (σ, Sigma). The clustering coefficient reflects the density of connections between each node and all of its neighbors. The shortest path length is the minimum number of pathways required to transfer information between any pair of nodes in the network, reflecting the efficiency of communication over a node’s direct neighbors. The small-world network is a model for describing complex brain networks with the criteria: γ > 1 and λ ≈ 1 or σ > 1 (σ = γ/λ) [18]. Small-world networks have higher Cp and similar Lp compared to random networks, indicating an optimal balance of functional integration and differentiation of the networks.

We proceeded to calculate the nodal metrics of the white matter structural networks, specifically degree centrality (DC), between centrality (BC), node efficiency (NE), and node local efficiency (NLE). DC measures the importance of a node or brain region in the overall brain network, whereas BC reflects the ability of a node to exert influence over the entire network. Furthermore, NE characterizes the efficiency of parallel information transfer by a node in the brain networks, while NLE quantifies how well a specific brain region can communicate with its adjacent regions, even if there are disruptions or damages to other regions or long-range connections in the network. Previous articles have provided a comprehensive and detailed description of these global and nodal metrics [19]. In this study, we used a sparsity threshold ranging from 0.05 to 0.5 with a step of 0.01 to analyze topological network properties to reduce the effect of bias caused by a single threshold [20]. The metrics of the structural network were calculated as the area under the curve (AUC). For the nodal metrics, the results were shown as corrected using the false discovery rate (FDR) with *p* < 0.05.

### 2.5. Network Based Statistics

White matter structural networks (FA-weighted networks in a symmetric 90 × 90 matrix) were analyzed using network-based statistics (NBS) toolbox, which performed permutation testing to assess differences in structural connectivity between groups [21]. Subnetworks with significant structural differences between groups were identified through multiple comparisons. We used a general linear model with 10,000 permutations and multiple comparisons correction (*p* = 0.05) to compare between-group connectivity. In NBS, correction for multiple comparisons was carried out via cluster-based thresholding whereby connected components of a network were treated as a cluster. We used the primary test statistic threshold (t = 2.1) to define a set of connections that exceeded the threshold because the corresponding *p*-value for connections with test statistic values exceeding this threshold was less than 0.05, indicating significance. The gender and age of all subjects were taken into account as relevant covariates in the NBS analysis. 

### 2.6. Statistical Analysis

Statistical analysis was performed in SPSS software (version 22.0; IBM SPSS Inc., Chicago, IL, USA). A two-sample *t*-test was used to assess the continuous variables, including age, MDVF, and RNFL thickness. Chi-square test was used to compare the differences in gender distribution between each NTG subgroup and the NC group. The global network matrices were compared between the NTG subgroup and the NC group using one-way analysis of variance (ANOVAs) and Dunnett’s post hoc test. Pearson correlation analysis was used to explore the association between mean MDVF and RNFL thickness and global network matrices in all NTG patients. The differences in regional properties between the two groups were analyzed using two-sample *t*-tests, and *p* < 0.05 was considered statistically significant.

## 3. Results

### 3.1. Demographic and Clinical Characteristics

The demographic characteristics and ophthalmologic examinations of the healthy controls and patients with NTG are presented in Table 1. No significant difference was found in the age and sex between the NC group and all of the NTG subgroups. RNFL thickness (*p* < 0.001, using Dunnett’s post hoc test) and MDVF (*p* < 0.001, using Dunnett’s post hoc test) were significantly lower in all NTG subgroups compared with the NC group.

### 3.2. Between-Group Difference in Global Topological Properties 

**Figure 1:**
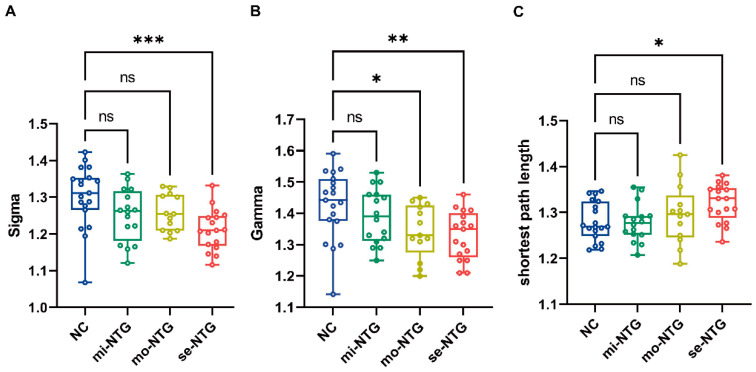
Box line chart of the Sigma, Gamma, and shortest path length between the NC and NTG subgroups. The comparisons between the NC and NTG subgroups showed a significant decrease in Sigma values for severe NTG, a decrease in Gamma values for moderate and severe NTG, and an increase in shortest path length for severe NTG. Sigma, the small world index; Gamma, normalized clustering coefficient; * *p* < 0.05, ** *p* < 0.01, *** *p* < 0.001, ns *p* > 0.05 (1-way ANOVA, Dunnett’s post hoc test).

**Figure 2:**
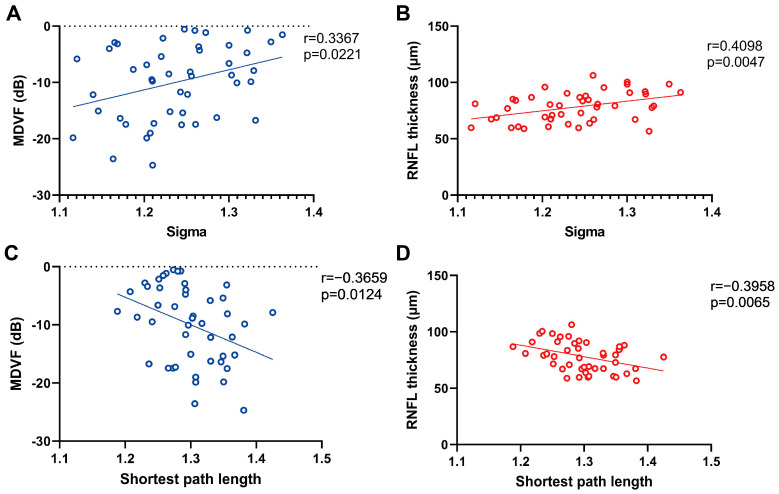
Scatter plots with regression lines showed the correlation between MDVF scores and Sigma (**A**), RNFL thickness and Sigma (**B**), MDVF scores and shortest path length (**C**), and RNFL thickness and shortest path length (**D**) in NTG patients. Sigma, the small world index; MDVF, mean deviation of visual field; RNFL, retinal nerve fiber layer.

As shown in Figure 1 and Table 2, compared with the NC group, the se-NTG subgroup demonstrated a significantly decreased the AUC of Sigma (*p* = 0.008) and increased AUC of shortest path length (*p* = 0.036) than the NC group. The AUCs of Gamma in mo-NTG (*p* = 0.038) and se-NTG subgroups (*p* = 0.008) were significantly decreased compared to the NC group. No significant differences were observed between the mi-NTG subgroups and NC groups. The AUC of sigma of NTG patients is positively correlated with the MDVF (r = 0.3367, *p* = 0.0221) and the RNFL (r = 0.4098, *p* = 0.0047). The AUC of the shortest path length of NTG patients is negatively correlated with the MDVF (r = −0.3659, *p* = 0.0124) and the RNFL (Figure 2) (r = −0.3958, *p* = 0.0065). 

### 3.3. Between-Group Difference in Local Topological Properties 

**Figure 3:**
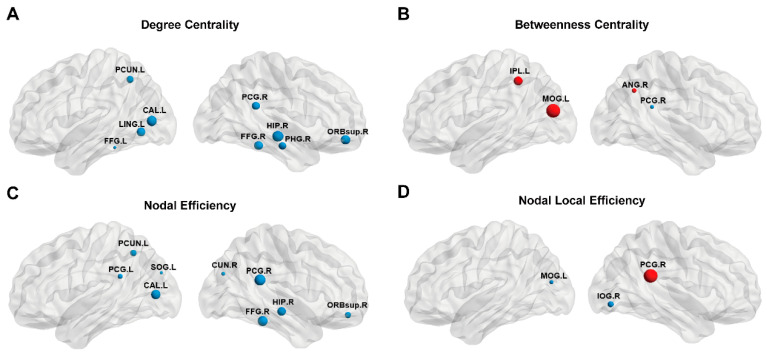
Three-dimensional view of brain hubs with differences between healthy controls and NTG patients. The brain map shows specific regions whose nodal property values including degree centrality (**A**), nodal efficiency (**B**), betweenness centrality (**C**), and nodal local efficiency (**D**) are higher (red) or lower (blue) in patients with NTG compared with controls. Node size: T-values for differences in nodal properties between the NTG group and healthy controls.

We found brain regions that showed significant between-groups differences in degree centrality, nodal efficiency, betweenness centrality, and nodal local efficiency. Compared with HCs, NTG patients showed significantly decreased degree centrality in the right superior frontal gyrus, orbital part (ORBsup.R), right parahippocampal gyrus (PHG.R), right fusiform gyrus (FFG.R), right hippocampus (HIP.R) left fusiform gyrus (FFG.L) right posterior cingulate gyrus (PCG.R), left precuneus (PCUN.L), left calcarine fissure and surrounding cortex (CAL.L) and left lingual gyrus (LING.L) (Figure 3A). Meanwhile, the nodal efficiency of ORBsup.R, FFG.R, HIP.R, PCG.L, PCG.R, PCUN.L, CAL.L, left superior occipital gyrus (SOG.L), right cuneus (CUN.R) decreased in NTG patients (Figure 3B). The betweenness centrality of the left inferior parietal, but supramarginal and angular gyri (IPL.L), right angular gyrus (ANG.R), and left middle occipital gyrus (MOG.L) in NTG were significantly increased, whereas the betweenness centrality of PCG.R in NTG was significantly reduced (Figure 3C). Compared with the HCs, NTG patients exhibited an increase in the local efficiency of the PCG. R along with a decrease in the local efficiency of MOG. L and the right inferior occipital gyrus (IOG.R) (Figure 3D) (All *p* < 0.05, FDR corrected).

### 3.4. Between-Group Difference in Structural Connectivity Revealed by NBS Analysis

**Figure 4:**
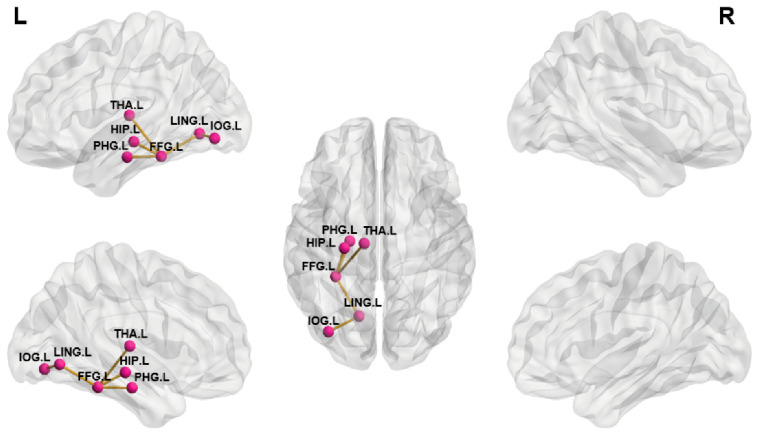
Three-dimensional view showing differences in structural connectivity between NTG patients and healthy controls. Edges determined using network-based statistics (NBSs) describe the stronger structural connectivity in the brain network of NTG patients.

As shown in Figure 4, the subnetworks that showed significant differences between groups were identified. Compared to HCs, NTG patients showed significantly stronger structural connectivity in a subnetwork comprising six brain regions and five connections. This subnetwork involved the left thalamus (THA.L), HIP.L, PHG.L, FFG.L, LING.L, and IOG.L. All of the above brain regions are directly connected to the FFG.L, except for the IOG.L, which is connected to the LING.L. 

## 4. Discussion

This study applied diffusion tensor tractography to evaluate the altered topological properties of structural brain networks in NTG patients. The main findings of the current study were as follows: (1) the small-world properties of white matter were disrupted in NTG patients and correlated with clinical indicators; (2) local topological properties of various brain regions were altered in NTG patients; and (3) structural connectivity of the occipitotemporal area was enhanced in NTG patients. These findings supported the hypothesis that white matter structural networks were abnormal in NTG patients.

In the present study, we found that both NTG and HC groups exhibited higher clustering coefficients and shorter shortest path lengths in the white matter structural networks, and both had efficient small-world characteristics (σ > 1). The clustering coefficient reflects the degree of modularity of the network; the higher the degree of modularity, the stronger the ability of brain function differentiation. The shortest path length measures the efficiency of network information transmission; the higher the efficiency of information transmission, the stronger the brain’s functional integration [22,23]. The combination of high local clustering and short path length possessed by the small-world feature supports two important organizational patterns in the brain and, therefore, enables efficient brain function: functional segregation and functional integration [24]. In the present study, compared with the control group, the shortest path length of the se-NTG group increased, while the normalized clustering coefficients of both the mo-NTG and the se-NTG groups decreased, corresponding to a significant decrease in the small-world index of the se-NTG group. The results demonstrated that the whole-brain functional integration in the structural network of NTG patients was disturbed [19], reflecting the widespread neurodegeneration across the brain in NTG [3]. In addition, the degree of visual impairment in NTG patients was negatively correlated with the shortest path length and positively correlated with the small-world index, suggesting that small-world properties were constantly being disrupted with the progression of retinal damage and visual field deficits [11]. Therefore, our findings indicated that white matter structural network properties may be a potential indicator for assessing NTG progression [25]. In addition, the correlation of small-world properties with ophthalmic impairment might support the notion that degeneration of NTG was involved with a brain factor [4], and thus, our study contributes to the idea that neuroprotective drugs to prevent the degeneration of brain structures in addition to IOP control may be beneficial in the treatment of NTG [26].

Our study showed that degree centrality, nodal efficiency, betweenness centrality, and nodal local efficiency were all altered in the white matter structural network of NTG patients. The degree centrality of CAL.L, FFG.R, FFG.L, and LING.L in the visual network of NTG patients was reduced, indicating a decrease in the number of other brain regions connected to the above nodes [27]. In addition, the nodal efficiency of the SOG.L, FFG.R, CAL.L, and CUN.R in the visual network was reduced, indicating a decrease in the efficiency of information transmission in these brain regions [27]. In particular, the nodes disrupted were mainly located in the ventral visual pathway. In contrast to the dorsal stream, which perceives “where”, the ventral stream perceives “what” and is responsible for form recognition [28]. This might explain the impairment of form perception in glaucoma patients [29]. In addition, the degree centrality and nodal efficiency of both ORBsup.R and HIP.R were reduced in NTG patients, demonstrating that information transmission in these brain regions was disturbed [30]. ORBsup.R is an important part of the limbic system, involved in self-regulation during emotional stress [31], whereas HIP.R is a key structure for spatial cognitive functions [32]. Additionally, abnormal nodal properties in ORBsup.R and HIP.R were reported to be associated with anxiety in primary insomnia patients [33]. Thus, the disruption of these nodes in the present study might reflect cognitive–emotional processing dysfunction, which is consistent with the previous study in glaucoma [34], suggesting that abnormalities of nodal topological properties in these regions might be an underlying neurobiological mechanism for the high prevalence of cognitive and emotional disorders in NTG [35,36,37]. 

We found elevated betweenness centrality of IPL and ANG in NTG patients, reflecting the enhanced ability of these nodes to act as mediators in communicating with other brain regions [38]. IPL and ANG are important components of the heteromodal association area, responsible for sensory integration, and, in particular, are involved in visuoproprioceptive realignment [39,40] and spatial cognition [41]. Therefore, in our study, the elevated betweenness centrality of IPL and ANG derived from graph theory reflected the enhanced integration of sensory functions in NTG, which coincided with previous studies in POAG [42,43], suggesting that improved multisensory integration in these regions might be an indicator of cross-modal plasticity that would compensate for visual field defects [44,45] in NTG. We also observed reduced local efficiency of MOG. L and IOG. R in NTG patients. Local efficiency could be interpreted as an indicator of system fault tolerance [46], and therefore, the decrease in local efficiency exhibited by NTG patients reflects the low resilience to damage in the visual network system of NTG patients, which might explain the connectivity disruption could be detected in the occipital lobe since the early disease stage [47].

Furthermore, in the NBS analysis of white matter structural networks, it was found that NTG patients had increased connectivity in the occipitotemporal area (occipital cortex, lingual gyrus, syringoid gyrus, and parahippocampal gyrus), with the left FFG as the hub compared to controls. Interestingly, a decrease in the degree centrality of the FFG was found in the nodal analysis of the current study, suggesting a decrease in the number of brain regions connected to the left FFG in NTG patients. The above evidence revealed an increased separation arising from the reconfiguration of the structural network in NTG patients, characterized by reduced global integration but increased connectivity within the subnetwork [24]. The left FFG is located in the occipitotemporal lobe and plays a key role in the lexical processing of reading, especially responsible for word form recognition [48,49,50]. These have been found to be positively associated with increased connectivity in the left occipitotemporal area [51]. Thus, enhanced connectivity within the left occipitotemporal region might reflect reading dysfunction, which coincided with previous studies in blind patients [52], indicating that abnormalities in this region may be a neuroplastic alteration contributing to compensatory performance after visual impairment. This shed light on the possibility that modulating cortical plasticity in the left occipitotemporal might be a beneficial strategy to activate the potential for high-order visual function recovery [53], as, for instance, non-invasive brain stimulation (NIBS) of the ventral visual pathway facilitates the recovery of reading ability [54].

Several shortcomings remain in this study. Firstly, due to the further division of patients into three subgroups, the small sample size of each subgroup restricted the statistical analysis at the subgroup level. Thus, future study with a larger sample size is necessary for further validation. Second, although our study observed associations between NTG clinical indicators and structural network alterations, whether these network topological changes in the brain are reversible requires in-depth studies using longitudinal designs. Finally, due to the hidden onset of NTG, we were unable to obtain the exact duration of the disease in patients. Moreover, the lack of information on participants’ educational level and cognitive abilities limits the further interpretation of our findings.

## 5. Conclusions

In summary, the small-world properties of white matter structural networks were disrupted in NTG patients compared to healthy controls and correlated with MDVF and RNFL thickness. Structural network indicators are expected to aid ophthalmologic examination for the course evaluation and monitoring of global neurodegeneration in NTG. In addition, the local topological properties of NTG patients in visual and nonvisual brain areas were also significantly different from those of healthy controls, with increased structural connectivity observed in the occipitotemporal area of NTG patients, which might be the potential neurobiological mechanism for visual and cognitive-emotional dysfunction in NTG. However, the lack of cognitive–emotional information limits the further interpretation of the results, which calls for the inclusion of more clinical information and behavioral tests in future studies. 

## Figures and Tables

**Table 1:** Comparisons of clinical information in normal controls as well as the NTG group and subgroups.

Characteristics	NC, n = 19	NTG, n = 46	mi-NTG, n = 16	mo-NTG, n = 13	se-NTG, n = 17	*p* Value	
						NC vs. NTG	NC vs. NTG Subgroups
Age, y	49.1 ± 13.5	50.0 ± 13.9	44.0 ± 11.6	51.5 ± 12.7	54.5 ± 15.3	0.806	ns
Male/female	10/9	26/20	9/7	7/6	10/7	0.774	ns
MDVF, dB							
Left	−1.2 ± 0.9	−9.3 ± 6.7	−3.4 ± 2.5	−9.1 ± 3.5	−15.1 ± 6.5	<0.001	<0.001
Right	−1.3 ± 0.7	−10.5 ± 8.4	−2.5 ± 1.9	−8.4 ± 4.6	−19.7 ± 4.8	<0.001	<0.001
Mean bilateral eyes	−1.2 ± 0.5	−9.9 ± 6.7	−2.9 ± 1.7	−8.8 ± 1.4	−17.4 ± 3.3	<0.001	<0.001
RNFL thickness, μm							
Left	103.2 ± 9.7	78.1 ± 13.1	85.3 ± 10.1	75.5 ± 11.5	73.5 ± 14.5	<0.001	<0.001
Right	102.3 ± 10.5	78.2 ± 17.8	89.1 ± 13.7	81.5 ± 21.0	65.3 ± 8.8	<0.001	<0.001
Mean bilateral eyes	102.7 ± 7.6	78.2 ± 13.1	87.2 ± 9.6	78.5 ± 14.2	69.4 ± 9.2	<0.001	<0.001

*p* values were calculated using Student’s *t*-test for NC versus NTG group and 1-way ANOVA for NC versus NTG subgroups (NC versus mi-NTG, mo-NTG, se-NTG, respectively) (Dunnett’s post hoc test is further used for MDVF and RNFL thickness); *p* values for the gender distribution were obtained using chi-square test; ns, *p* > 0.05; MDVF, mean deviation of visual field; RNFL, retinal nerve fiber layer.

**Table 2:** Comparisons of the global topological properties of the white matter structural networks in normal controls as well as the NTG group and subgroups.

Measure	NC	NTG	mi-NTG	mo-NTG	se-NTG	*p* Value			
						NC vs. NTG	NC vs. mi-NTG	NC vs. mo-NTG	NC vs. se-NTG
Sigma	1.30 ± 0.09	1.24 ± 0.06	1.26 ± 0.06	1.23 ± 0.06	1.22 ± 0.06	0.002 **	0.227	0.018 *	0.003 **
Lambda	0.53 ± 0.01	0.53 ± 0.01	0.53 ± 0.01	0.53 ± 0.01	0.53 ± 0.01	0.054	0.976	0.102	0.057
Gamma	1.43 ± 0.11	1.40 ± 0.09	1.34 ± 0.09	1.33 ± 0.08	1.36 ± 0.09	0.007 **	0.546	0.038 *	0.008 **
Lp	1.28 ± 0.04	1.30 ± 0.05	1.28 ± 0.04	1.30 ± 0.07	1.32 ± 0.04	0.168	0.998	0.683	0.036 *
Cp	0.22 ± 0.01	0.23 ± 0.01	0.23 ± 0.01	0.23 ± 0.01	0.22 ± 0.01	0.132	0.369	0.534	0.628

All values of global topological properties were expressed as the area under the curve (AUC); *p* values were calculated by using Student’s *t*-test for NC versus NTG group (* *p* < 0.05) and 1-way ANOVA with Dunnett’s post hoc test for NC versus NTG subgroups (* *p* < 0.05; ** *p* < 0.01); Sigma, the small world index; Lambda, normalized shortest path length; Gamma, normalized clustering coefficient; Lp, shortest path length; Cp, clustering coefficient.

## Data Availability

The data are available upon reasonable request.

## References

[1] Gittinger J.W. (2019). Management of normal tension glaucoma. Surv. Ophthalmol..

[2] Zhang H.J., Mi X.S., So K.F. (2019). Normal tension glaucoma: From the brain to the eye or the inverse?. Neural Regen. Res..

[3] Giorgio A., Zhang J., Costantino F., De Stefano N., Frezzotti P. (2018). Diffuse brain damage in normal tension glaucoma. Hum. Brain Mapp..

[4] Boucard C.C., Hanekamp S., Curcic-Blake B., Ida M., Yoshida M., Cornelissen F.W. (2016). Neurodegeneration beyond the primary visual pathways in a population with a high incidence of normal-pressure glaucoma. Ophthalmic Physiol. Opt..

[5] Afzali M., Pieciak T., Newman S., Garyfallidis E., Ozarslan E., Cheng H., Jones D.K. (2021). The sensitivity of diffusion MRI to microstructural properties and experimental factors. J. Neurosci. Methods.

[6] Schmidt M.A., Knott M., Heidemann R., Michelson G., Kober T., Dorfler A., Engelhorn T. (2018). Investigation of lateral geniculate nucleus volume and diffusion tensor imaging in patients with normal tension glaucoma using 7 tesla magnetic resonance imaging. PLoS ONE.

[7] Wang R., Tang Z.H., Sun X.H., Wu L.J., Wang J., Zhong Y.F., Xiao Z.B. (2018). White Matter Abnormalities and Correlation With Severity in Normal Tension Glaucoma: A Whole Brain Atlas-Based Diffusion Tensor Study. Investig. Ophthalmol. Vis. Sci..

[8] Sporns O. (2018). Graph theory methods: Applications in brain networks. Dialogues Clin. Neurosci..

[9] Wang J.Q., Li T., Wang N.L., Xian J.F., He H.G. (2016). Graph theoretical analysis reveals the reorganization of the brain network pattern in primary open angle glaucoma patients. Eur. Radiol..

[10] Minosse S., Garaci F., Martucci A., Lanzafame S., Di Giuliano F., Picchi E., Cesareo M., Mancino R., Guerrisi M., Floris R. Disruption of brain network organization in primary open angle glaucoma. Proceedings of the 2019 41st Annual International Conference of the IEEE Engineering in Medicine and Biology Society (EMBC).

[11] Di Cio F., Garaci F., Minosse S., Passamonti L., Martucci A., Lanzafame S., Di Giuliano F., Picchi E., Cesareo M., Guerrisi M.G. (2020). Reorganization of the structural connectome in primary open angle Glaucoma. NeuroImage Clin..

[12] Yang F., Zhang J., Fan L., Liao M., Wang Y., Chen C., Zhai T., Zhang Y., Li L., Su L. (2020). White matter structural network disturbances in first-episode, drug-naive adolescents with generalized anxiety disorder. J. Psychiatr. Res..

[13] Cui Z., Zhong S., Xu P., He Y., Gong G. (2013). PANDA: A pipeline toolbox for analyzing brain diffusion images. Front. Hum. Neurosci..

[14] Basser P.J., Pajevic S., Pierpaoli C., Duda J., Aldroubi A. (2000). In vivo fiber tractography using DT-MRI data. Magn. Reson. Med..

[15] Yuan J.P., Henje Blom E., Flynn T., Chen Y., Ho T.C., Connolly C.G., Dumont Walter R.A., Yang T.T., Xu D., Tymofiyeva O. (2019). Test-Retest Reliability of Graph Theoretic Metrics in Adolescent Brains. Brain Connect..

[16] Shu N., Wang X., Bi Q., Zhao T., Han Y. (2018). Disrupted Topologic Efficiency of White Matter Structural Connectome in Individuals with Subjective Cognitive Decline. Radiology.

[17] Wang J., Wang X., Xia M., Liao X., Evans A., He Y. (2015). GRETNA: A graph theoretical network analysis toolbox for imaging connectomics. Front. Hum. Neurosci..

[18] Uehara T., Yamasaki T., Okamoto T., Koike T., Kan S., Miyauchi S., Kira J., Tobimatsu S. (2014). Efficiency of a “small-world” brain network depends on consciousness level: A resting-state FMRI study. Cereb. Cortex.

[19] Rubinov M., Sporns O. (2010). Complex network measures of brain connectivity: Uses and interpretations. NeuroImage.

[20] Liu Y., Li F., Shang S., Wang P., Yin X., Krishnan Muthaiah V.P., Lu L., Chen Y.C. (2023). Functional-structural large-scale brain networks are correlated with neurocognitive impairment in acute mild traumatic brain injury. Quant. Imaging Med. Surg..

[21] Zalesky A., Fornito A., Bullmore E.T. (2010). Network-based statistic: Identifying differences in brain networks. NeuroImage.

[22] Iturria-Medina Y., Sotero R.C., Canales-Rodriguez E.J., Aleman-Gomez Y., Melie-Garcia L. (2008). Studying the human brain anatomical network via diffusion-weighted MRI and Graph Theory. NeuroImage.

[23] He Y., Evans A. (2010). Graph theoretical modeling of brain connectivity. Curr. Opin. Neurol..

[24] Cohen J.R., D’Esposito M. (2016). The Segregation and Integration of Distinct Brain Networks and Their Relationship to Cognition. J. Neurosci..

[25] Lalezary M., Medeiros F.A., Weinreb R.N., Bowd C., Sample P.A., Tavares I.M., Tafreshi A., Zangwill L.M. (2006). Baseline optical coherence tomography predicts the development of glaucomatous change in glaucoma suspects. Am. J. Ophthalmol..

[26] Nucci C., Strouthidis N.G., Khaw P.T. (2013). Neuroprotection and other novel therapies for glaucoma. Curr. Opin. Pharmacol..

[27] Sporns O., Honey C.J., Kotter R. (2007). Identification and classification of hubs in brain networks. PLoS ONE.

[28] Wurm M.F., Caramazza A. (2022). Two ‘what’ pathways for action and object recognition. Trends Cogn. Sci..

[29] McKendrick A.M., Badcock D.R., Morgan W.H. (2005). The detection of both global motion and global form is disrupted in glaucoma. Investig. Ophthalmol. Vis. Sci..

[30] Bullmore E., Sporns O. (2009). Complex brain networks: Graph theoretical analysis of structural and functional systems. Nat. Rev. Neurosci..

[31] Dixon M.L., Thiruchselvam R., Todd R., Christoff K. (2017). Emotion and the prefrontal cortex: An integrative review. Psychol. Bull..

[32] Vikbladh O.M., Meager M.R., King J., Blackmon K., Devinsky O., Shohamy D., Burgess N., Daw N.D. (2019). Hippocampal Contributions to Model-Based Planning and Spatial Memory. Neuron.

[33] Wu Y., Liu M., Zeng S., Ma X., Yan J., Lin C., Xu G., Li G., Yin Y., Fu S. (2018). Abnormal Topology of the Structural Connectome in the Limbic Cortico-Basal-Ganglia Circuit and Default-Mode Network among Primary Insomnia Patients. Front. Neurosci..

[34] Frezzotti P., Giorgio A., Motolese I., De Leucio A., Iester M., Motolese E., Federico A., De Stefano N. (2014). Structural and functional brain changes beyond visual system in patients with advanced glaucoma. PLoS ONE.

[35] Yochim B.P., Mueller A.E., Kane K.D., Kahook M.Y. (2012). Prevalence of cognitive impairment, depression, and anxiety symptoms among older adults with glaucoma. J. Glaucoma.

[36] Cui Q.N., Green D., Jethi M., Driver T., Porco T.C., Kuo J., Lin S.C., Stamper R.L., Han Y., Chiu C.S. (2021). Individuals with and without normal tension glaucoma exhibit comparable performance on tests of cognitive function. Int. J. Ophthalmol..

[37] Zhang D., Fan Z., Gao X., Huang W., Yang Q., Li Z., Lin M., Xiao H., Ge J. (2018). Illness uncertainty, anxiety and depression in Chinese patients with glaucoma or cataract. Sci. Rep..

[38] Liu Q., Shi Z., Wang K., Liu T., Funahashi S., Wu J., Zhang J. (2022). Treatment Enhances Betweenness Centrality of Fronto-Parietal Network in Parkinson’s Patients. Front. Comput. Neurosci..

[39] Sack A.T. (2009). Parietal cortex and spatial cognition. Behav. Brain Res..

[40] Seghier M.L. (2013). The angular gyrus: Multiple functions and multiple subdivisions. Neuroscientist.

[41] Sirigu A., Duhamel J.R., Cohen L., Pillon B., Dubois B., Agid Y. (1996). The mental representation of hand movements after parietal cortex damage. Science.

[42] Wang J., Li T., Sabel B.A., Chen Z., Wen H., Li J., Xie X., Yang D., Chen W., Wang N. (2016). Structural brain alterations in primary open angle glaucoma: A 3T MRI study. Sci. Rep..

[43] Li T., Liu Z., Li J., Liu Z., Tang Z., Xie X., Yang D., Wang N., Tian J., Xian J. (2014). Altered amplitude of low-frequency fluctuation in primary open-angle glaucoma: A resting-state FMRI study. Investig. Ophthalmol. Vis. Sci..

[44] Rauschecker J.P., Tian B., Korte M., Egert U. (1992). Crossmodal changes in the somatosensory vibrissa/barrel system of visually deprived animals. Proc. Natl. Acad. Sci. USA.

[45] Rauschecker J.P. (1995). Compensatory plasticity and sensory substitution in the cerebral cortex. Trends Neurosci..

[46] Latora V., Marchiori M. (2001). Efficient behavior of small-world networks. Phys. Rev. Lett..

[47] Frezzotti P., Giorgio A., Toto F., De Leucio A., De Stefano N. (2016). Early changes of brain connectivity in primary open angle glaucoma. Hum. Brain Mapp..

[48] Tsapkini K., Rapp B. (2010). The orthography-specific functions of the left fusiform gyrus: Evidence of modality and category specificity. Cortex.

[49] Cohen L., Lehericy S., Chochon F., Lemer C., Rivaud S., Dehaene S. (2002). Language-specific tuning of visual cortex functional properties of the Visual Word Form Area. Brain.

[50] Cohen L., Dehaene S., Naccache L., Lehericy S., Dehaene-Lambertz G., Henaff M.A., Michel F. (2000). The visual word form area—Spatial and temporal characterization of an initial stage of reading in normal subjects and posterior split-brain patients. Brain.

[51] Del Mauro G., Del Maschio N., Abutalebi J. (2022). The relationship between reading abilities and the left occipitotemporal sulcus: A dual perspective study. Brain Lang..

[52] Liu Y., Yu C., Liang M., Li J., Tian L., Zhou Y., Qin W., Li K., Jiang T. (2007). Whole brain functional connectivity in the early blind. Brain.

[53] Turker S., Hartwigsen G. (2021). Exploring the neurobiology of reading through non-invasive brain stimulation: A review. Cortex.

[54] Arrington C.N., Ossowski A.E., Baig H., Persichetti E., Morris R. (2023). The Impact of Transcranial Magnetic Stimulation on Reading Processes: A Systematic Review. Neuropsychol. Rev..

